# Iatrogenic Benign Subcutaneous Emphysema of the Left Upper Limb

**DOI:** 10.7759/cureus.22808

**Published:** 2022-03-03

**Authors:** Snehasis Das, Oseen Shaikh, Naveen K Gaur, Gopal Balasubramanian

**Affiliations:** 1 Surgery, Jawaharlal Institute of Postgraduate Medical Education and Research, Puducherry, IND

**Keywords:** crepitus, compartment syndrome, air gun, pressurized air, subcutaneous emphysema

## Abstract

Subcutaneous emphysema refers to the presence of air in the subcutaneous planes of the body. It may result from a benign cause like trauma, accidental injection, or entry of air through a negative pressure gradient, or it could be a part of the life-threatening ailment in the form of necrotizing fasciitis with gas gangrene. We report a 31-year-old male who sustained trauma to the hand followed by pressurized air injection into the wound resulting in the subcutaneous emphysema of the left upper limb. Imaging studies confirmed the presence of subcutaneous emphysema. The patient was managed conservatively with limb immobilization. Being rarely reported in the medical literature, we aim to improve the awareness of such a condition and beware of impending complications.

## Introduction

Subcutaneous emphysema of the upper limb has rarely been reported in the medical literature. It can be caused due to both infectious and non-infectious cases with completely varied treatment and outcomes for which rapid diagnosis is a dictum to prevent avoidable mishaps. The non-infectious benign type has been known to be caused due to a variety of causes, while the infectious necrotizing fasciitis or gas gangrene can eventually be lethal [1,2]. Benign subcutaneous emphysema usually presents with subtle symptoms of air under the tissues and can rarely present with features of compartment syndrome. History and clinical examination aid in the diagnosis of the condition. Imaging is done to identify air under the subcutaneous tissue objectively. Treatment is usually conservative, and rarely is surgical intervention required. Herein we describe a case of benign subcutaneous emphysema, which developed following self-injection of pressurized air following a small injury over the hand.

## Case presentation

**Figure 1:**
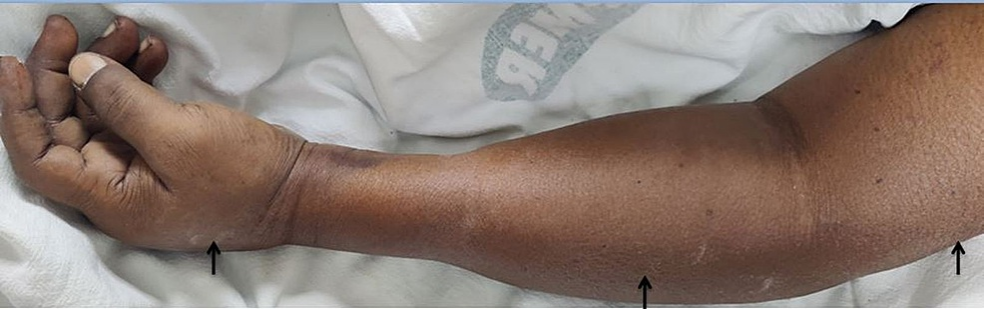
Clinical picture of the left upper limb showing subcutaneous emphysema (arrows)

A 31-year-old male, without any known medical comorbidities, presented with an alleged history of trauma to the left hand in his workplace with minimal blood ooze. The patient attempted to clean the blood with the help of a pressurized air gun leading to the injection of air into the left upper limb. Following this, the patient had acute onset swelling of the left hand and left forearm with proximal extension. It was not associated with any pain, restriction of movements, or sensory synesthesia. On examination, he was well built and nourished. The patient’s vitals were stable, with a blood pressure of 120/70 mmHg and a pulse rate of 76 beats per minute. Examination of other systems was within normal limits. Local examination of the left upper limb was suggestive of swelling in the left hand and forearm, with crepitus being felt up to the distal thirds of the forearm (Figure 1).

A linear puncture of 0.5 cm was seen in the first digital web space in the left hand with no features of ascending cellulitis. Distal pulses were palpable with no features suggestive of an impending compartment syndrome.

**Figure 2:**
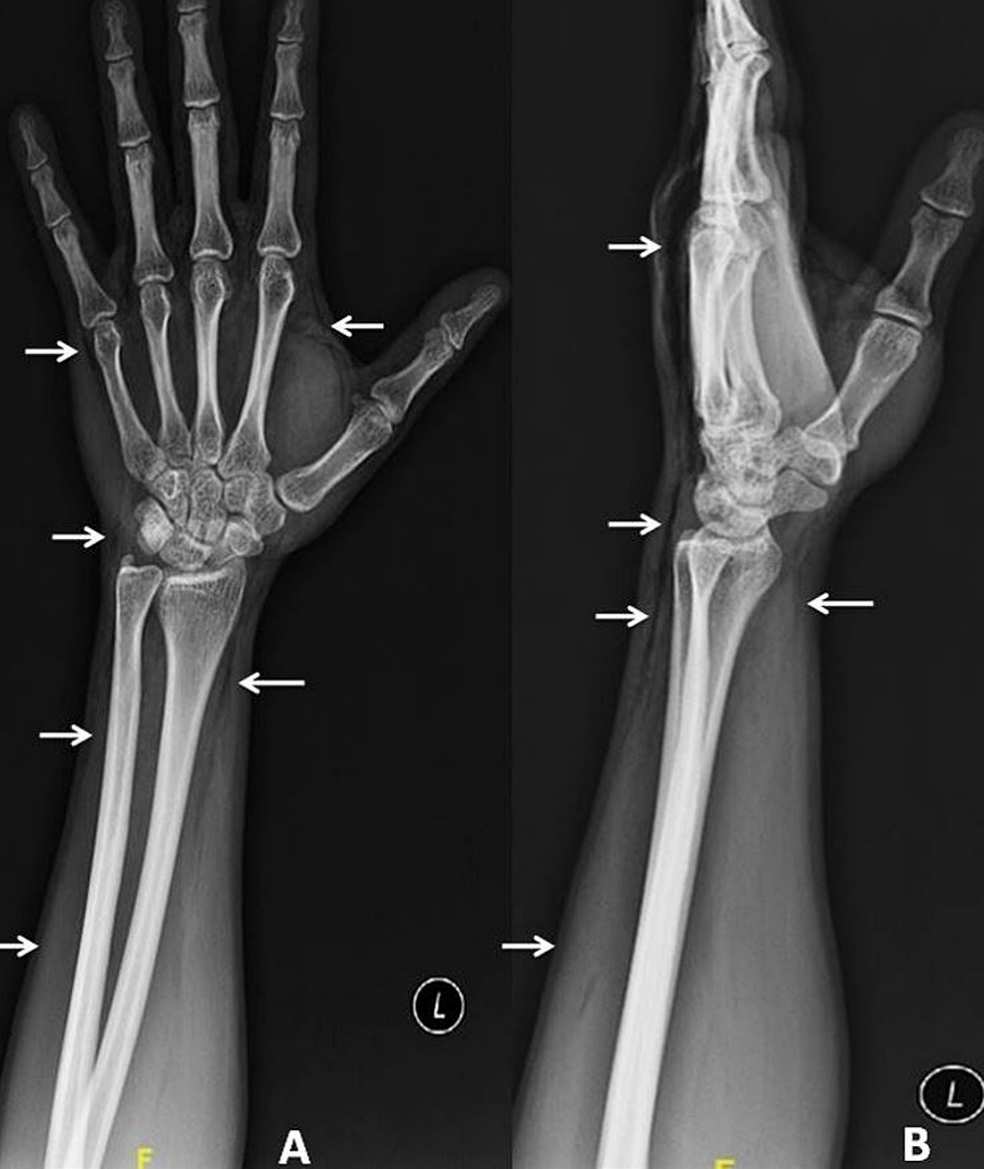
X-ray of the left upper limb showing subcutaneous emphysema A: anteroposterior view, B: lateral view

Blood investigations showed normal hemoglobin, and white blood cell (WBC) counts. Renal function tests and liver function tests were normal. X-ray of the left upper limb was suggestive of subcutaneous air in the left arm and forearm compartments. There was no evidence of any fractures or other injuries (Figure 2).

Venous doppler of the left upper limb and neck veins was done, and there was no evidence of any venous thrombus or proximal venous outflow obstruction. The patient was stable, hence he was planned for conservative management. The patient was given a tight compression dressing to the left upper limb. The patient was reassured and followed up after two weeks, which marked complete resolution of the crepitus with no signs of limb functionality compromise.

## Discussion

Benign subcutaneous emphysema of the upper limb is rarely documented in the medical literature. It has been seen to arise from both infectious and non-infectious causes, with the infectious spectrum bearing aggressive and malefic lethality. On the other hand, the benign causes are usually managed conservatively, and prompt treatment usually resolves the ailment without any limb-threatening complication. Therefore, it is paramount to differentiate between the two and give the appropriate treatment.

The proposed theory behind the pathomechanism lies in the “ball valve type mechanism” when air is sucked in through a discontinuity in the skin [2,3]. The same was seconded by Crampton, who additionally proposed that the bandage and the application of the bandage with immobilization of the affected limb create the said mechanism [4]. The non-infectious causes of benign subcutaneous emphysema have been documented to range from migration of an internal orthopedic nail fixation to pneumatic tools. It has also been rarely reported after dental procedures, air gun injection, self-harm, and insect bite [1-3].

Although it has been seen that the handful of the documented cases have been treated conservatively and have resulted in spontaneous resolution with minimal intervention, it is of utmost importance to differentiate from a subclinical stage of developing myonecrosis or necrotizing fasciitis. The infective causes are usually post-traumatic and precipitated by gas-forming organisms, including clostridia, anaerobic streptococcus, or rare coliform bacteria. The diagnosis has to be apt as it calls for immediate and aggressive surgical ministrations in the form of wound debridement, amputation compounded with high-end antibiotics, and hyperbaric oxygen therapy [5].

The diagnosis of such cases requires a thorough history taking and clinical examination, which reveals the crepitus. Most of the cases exhibit only minimal pain and swelling with the absence of extensive cellulitis or signs of systemic sepsis. The primary wound is treated based on the size, site, and amount of contamination which heralds local antisepsis in irrigation wound dressing and a course of antibiotics. The subcutaneous air in the affected area is seen to be self-absorbed into the tissues over one to three weeks, depending on the volume and site of emphysema. As a form of caution, the affected area should be immobilized at the proximal joint to prevent the ball-valve entry of air, as proposed by Crampton [4].

The only scenario of a fatal outcome in such a benign ailment is in the form of compartment syndrome caused due to the air inside the anatomical compartments. A reading of delta pressure less than 30 mmHg is supposed to cause impedance in the microcapillary circulation, which causes ischemic necrosis of the compartmental tissues [6]. A pressure below 20 mmHg indicates immediate compartmental fasciotomy to relieve the pressure, or it might lead to permanent damage. All patients, therefore, should be thoroughly examined, and the clinician should look out for impending signs of compartment syndrome, which includes pain, especially on passive stretching, pallor, poikilothermia, pulselessness, paralysis, and paresthesia.

The patient was clinically stable with no signs of local or systemic complications in our case. The wound was treated with thorough irrigation of the wound and oral antibiotics. The arm was immobilized with a sling, and the patient was followed up after two weeks, which resulted in the complete resolution of the emphysema with no additional functionality loss.

## Conclusions

Benign subcutaneous emphysema has to be differentiated from its close counterparts, such as gas gangrene, which usually harbor a stormy clinical progression and leads to eventual mortality. A detailed history and clinical examination aids in the rapid diagnosis and requires a conservative form of management. Nevertheless, the emphysema might complicate a compartment syndrome in the affected site resulting in a catastrophic prognosis, and hence clinicians should keep an eye out for it. Spontaneous resolution is the usual course, as in our case, but emergency surgical decompression might be warranted in a handful of cases.
